# The impact of obesity on chronic oedema/lymphoedema of the leg – an international multicenter cross-sectional study (LIMPRINT)

**DOI:** 10.1038/s41366-024-01544-0

**Published:** 2024-07-03

**Authors:** Ewa Anna Burian, Jørgen Rungby, Tonny Karlsmark, Susan Nørregaard, Marina Cestari, Peter J. Franks, Christine Joy Moffatt

**Affiliations:** 1 Department of Dermato-Venereology & Wound Healing Centre, Bispebjerg Hospital, Copenhagen University Hospital, Copenhagen, Denmark; 2 Steno Diabetes Center Copenhagen, Herlev, Denmark; 3 Pianeta Linfedema Study Centre, Terni, Italy; 4 Centre for Research and Implementation of Clinical Practice, London, United Kingdom; 5 Nottingham University Hospitals NHS Trust, Nottingham, United Kingdom

**Keywords:** Obesity, Inflammatory diseases, Epidemiology

## Abstract

**Background/Objectives:**

Obesity and chronic oedema/lymphoedema are two distinct but related conditions, rarely investigated together. The aim was to study the impact of increased weight on chronic oedema and related factors.

**Subjects/Methods:**

A cross-sectional study, 38 centers, nine countries. Patients with clinically confirmed chronic oedema/lymphoedema of the leg were included. Weight category was estimated as: normal weight (BMI 20–30), class I-II obesity (BMI 30–40), or class III obesity (BMI > 40). Factors were tested for an association with increased weight, using a multivariable model.

**Results:**

A total of 7397 patients were included; 43% with normal weight, 36% class I-II obesity and 21% class III obesity. Increased weight was associated with more advanced stages of chronic oedema (ISL stage III; the most advanced form); affecting 14% in normal weight, 18% class I-II obesity and 39% class III obesity (*p* < 0.001). Ten factors were independently associated with increased weight: diabetes (OR 2.4), secondary lymphoedema (OR 2.7), cellulitis/erysipelas within 12 months (OR 1.2), bilateral lymphoedema (OR 3.6), compression therapy (OR 2.1), increased swelling duration (1–2 years OR 1.3, 2–5 years OR 2.5, 5–10 years OR 3.6, >10 years OR 3.5) decreased mobility (walking with aid OR 1.9, being chair bound OR 1.2) and age (reference<45 years; 45–64 years OR 1.5, 75–84 years OR 0.6, 85+ years OR 0.2). Increased weight was associated with a lower presentation of peripheral arterial disease (OR 0.7) and poorer chronic oedema control (OR 0.8). Patients with obesity had lower function, appearance and more severe symptoms (LYMQOL) and lower quality of life (EuroQol).

**Conclusions:**

Obesity negatively impacts chronic oedema, leading to more advanced stages. Achieving good control of swelling with compression is more difficult in these patients. Increased awareness of chronic oedema/lymphoedema as a complication of obesity is important for early detection and for developing effective strategies to prevent and manage them.

## Introduction

The increasing prevalence of obesity and related comorbidities is draining health care resources across the world. Numerous comorbidities are associated with obesity including type 2 diabetes, hypertension, cardiovascular diseases and sleeping disorders [1]. Lately, obesity has been recognized as a cause of lymphoedema/chronic oedema [2–4] with a significant overrepresentation of lymphoedema in patients with obesity [5]. In a study of 330 patients with severe obesity 33% had a lymphoedema-like swelling [6].

Lymphoedema is a disease caused by either developmental issues (primary) or a destruction of the lymphatic system (secondary), e.g. by cancer obstruction/surgery, venous insufficiency, parasitic nematodes filariasis [7] or obesity. More than one cause of lymphoedema can be found [8] and other factors such as cardiac and/or renal insufficiency can also contribute to the oedema development. For this reason, the umbrella term chronic oedema has been introduced (oedema >3 months). Irrespective of the cause, these conditions lead to soft-tissue swelling, increasing the risk of bacterial cellulitis, movement restriction, functional loss, and reduced quality of life [7]. The stagnant lymphatic fluid in lymphoedema initiates an inflammatory response with T cell activation, and induction of Th2 cytokines IL-4 and Il-13 that along with TGF-*β*1 contribute to increased lymphatic leakiness, decreased lymphatic pumping and fibrosis development [9]. This is reflected clinically; in the initial phases, a soft swelling is developed with pitting oedema, and with time fibrosis, hyperkeratosis, dermal thickening and warty overgrowths develop, including fat tissue deposition [10, 11]. The stimulation of the adipogenesis in lymphoedema has been proven both in humans and animal models [12, 13].

Severe obesity is a well-established risk factor for secondary lymphoedema in the legs and arms following cancer surgery [14, 15]. However, an increased risk of lymphoedema in patients with obesity but without any surgery has lately been reported [2, 3, 16–18]. The risk of lymphatic dysfunction seem to be predicted by the BMI, being reported as 0% in patients with BMI below 40, 17% in BMI 40–49, 63% in BMI 50–59, and 100% in BMI above 80 [18].

These findings show that lymphoedema can cause fat tissue deposition, and obesity can cause lymphoedema; suggesting these conditions are deeply interlinked. Although obesity has been established as a cause of lymphoedema/chronic oedema, few researchers have attempted to characterize this patient population within an international study. The authors investigated the impact of increased obesity classes (normal weight, class I-II obesity, and class III obesity) on the clinical characteristics of chronic oedema/lymphoedema of the leg, and sought to investigate the size of the problem of obesity in these patients.

## Methods

### Study design

A prospective, international, multicenter, cross-sectional study performed between June 2014 and August 2017, as part of the LIMPRINT-project (acronym for Lymphedema IMpact and PRevalence INTernational) and was run under the charity International Lymphedema Framework. Every country and center achieved necessary approvals, including from the Ethical Review Committee. All methods were performed in accordance with relevant guidelines and regulations, including the Declaration of Helsinki. Written informed consent was secured at all centers, requiring this. The development and validation of the study has previously been published [19].

### Participants

Participants were recruited from both hospital settings and community health services, e.g. general hospitals, nursing homes, specialist lymphoedema clinics and wound care clinics.

Patients were included if having clinically determined chronic leg oedema (oedema over 3 months of duration, irrespective of the cause); confirmed by a positive pitting oedema test and/or a positive Stemmer’s sign. Patients provided informed consent and were older than 18 years of age. Excluding criteria were those receiving end-of-life treatment or evaluated by clinicians as not in the patient’s best interest. In the study patients being underweight (BMI < 20) were not included in the data analysis.

### Definitions

#### Chronic oedema

Determined in this study by either a positive pitting oedema test and/or a Stemmer’s sign. The oedema was judged as chronic if present for 3 months or more, based on anamnestic feedback from the patient, caregivers and clinicians knowing the patient for at least three months. The adapted pitting oedema test is positive if a pit in the skin remains after pressing the thumb for 10 s at the site of swelling with a further category for those with non-pitting oedema and skin changes. The Stemmer’s sign is positive if a skin fold at the base of the second toe cannot be pinched/lifted and indicated longstanding oedema with fibrosis in the tissue [19]. Patients were included irrespective of the cause(s) of chronic oedema. Some have argued that all chronic oedema involves an element of lymphoedema [20]. Centers with the appropriate expertise performed a further evaluation of the severity of chronic oedema/lymphoedema, according to the ISL-stages:

*ISL-stage I:* Early onset, with accumulation of tissue oedema that reduces with leg elevation. The oedema may be pitting.

*ISL-stage II:* Leg elevation alone rarely reduces the oedema, pitting is manifested.

*ISL-stage III:* Hard (fibrotic) tissue is present, while pitting is absent. Skin changes e.g. thickening, hyperpigmentation, increased skin folds, fat deposits, and warty skin is seen.

### Body weight categories

A WHO general category of weight was adopted due to the lack of available BMI for some patients, particularly those seen in community settings [19]. Body weight category was estimated as:

*Normal weight:* BMI 20–30; herein also including patients classified as having overweight/pre-obesity

*Class I to II obesity:* BMI 30–40 (thereby a joint classification of class I obesity BMI 30–35, and class II obesity BMI 35–40) or

*Class III obesity:* BMI > 40.

### Data collected and quality assurance

Data was collected by health care professionals using the CoreTool questionnaire which has previously been published. The tool includes 13 domains: type of facility in which data are collected, demographics, level of obesity, mobility, relevant comorbidities, classification of lymphoedema and assumed causes, oedema history, cellulitis history, categories of treatment, site of swelling, wounds, access to treatment, and subjective control of swelling – the latter being judged by the investigator. All investigators were trained, both with written standards and a video was made to demonstrate how to undertake the pitting test, recognition of a positive Stemmer’s sign, and fibrosis. Data collection and quality assurance has been covered elsewhere [19].

### Quality of life

Two tools were used to assess health related quality of life; LYMQOL a disease specific tool measuring the impact of lymphoedema on the patient’s everyday living and health-related quality of life, and EuroQol (EQ-5D), a generic tool applicable to a wide range of health conditions and provides a simple descriptive profile and single-index value for health status. Both tools were filled out by the patient themselves. LYMQOL covers 27 items in four domains: symptoms, body image/ appearance, function and mood. Each item is scored between 1 (not at all) and 4 (a lot) and a total score is calculated. Low values of LYMQOL indicate less impact on the patient, with higher scores demonstrating greater impact. The assessed overall quality of life in LYMQOL goes from zero (worst health) to 10 (best health). EuroQol (EQ-5D) to a wide range of health conditions and provides a simple descriptive profile and single-index value for health status. Five dimensions are measured: mobility, self-care, usual activities, pain/discomfort, and anxiety/depression. Each dimension has three levels: no problems, some problems, and extreme problems. For each dimension, the patient’s choice results in a one-digit number. A score 0 (death) to 1.00 (perfect health), with some health states giving a negative score (worse than death).

### Statistical methods

Being an explorative study, a formal sample size analysis was not performed. However, an excess of 7000 patients should be adequate to provide for a suitable power for analysis. The principal analysis was undertaken comparing a number of independent clinical variables with the levels of obesity as the dependent variable. For this analysis ordinal logistic regression was used which allows for more than two values for the dependent variable, assuming a proportional odds relationship between values. Determination of the independent factors within a multivariable model were undertaken using the ordinal logistic model with a stepwise elimination until all remaining variables had a *p* < 0.05. These were presented as proportional odds ratios with 95% confidence intervals given.

## Results

### Countries and study sites

Participants were recruited from 38 centers across Australia, Canada, Denmark, France, Ireland, Italy, Japan, Turkey and UK. The majority of patients were treated in specialist lymphoedema centers (75.9%), fewer were recruited from general hospital services (17.0%), community health services (1.6%) and other services (5.5%).

### Demographics

A total number of 7397 patients were included with chronic leg oedema; of which 43.3% (3205/7397) were classified as normal weight, 35.7% with class I or II obesity (2639/7397) and 21.0% with class III obesity (1552/7397). The percentage of patients with class III obesity greatly varied; from to 0.9% in Italy to 47.1% in Canada, Table 1. The mean age was 65.2 years of age with nearly three quarters being women (70.5%). A total of 81.0% of patients had secondary lymphoedema (5863/7241) while the rest had primary lymphoedema as defined by clinicians. Among patients with secondary lymphedema 17.5% were judged as related to cancer or its treatment, while the rest had non-cancer related oedema (82.5%); 49.2% due to venous diseases, 37.2% due to immobility, 30.7% due to obesity and 0.2% due to filariasis. Patients were able to have a number of co-contributing factors within the classification of their chronic oedema. Further demographic data is depicted in Table 2.

**Table 1:** Percentage of patients within each obesity class by country.

Country	*n*	Normal weight (BMI 20–30)	Class I and II obesity (BMI 30–40)	Class III obesity (BMI > 40)
Canada	68	19.12	33.82	47.06
UK	4649	29.64	40.87	29.49
Australia	97	32.99	39.18	27.84
Turkey	213	52.58	37.56	9.86
Denmark	812	62.81	28.82	8.37
Ireland	18	61.11	33.33	5.56
France	332	73.49	21.08	5.42
Japan	163	84.66	12.27	3.07
Italy	1045	73.49	25.65	0.86
Total	7397	43.34	35.68	20.99

Countries are ranked according to the percentage of patients who had class III obesity.

**Table 2:** Demographics of patients with chronic leg oedema (*n* = 7397).

Characteristic(s)	Number of patients (%)
Age, mean	65.17 (sd = 16.38)
Missing	3
Female	5214 (70.50)
Missing	1
Body weight	
Normal weight (BMI 20–30)	3205 (43.34)
Class I and II obesity (BMI 30–40)	2639 (35.68)
Class III obesity (BMI > 40)	1552 (20.98)
Missing	0
Facility	
Specialist lymphoedema service	5616 (75.92)
Non specialist hospital based service	1259 (17.02)
Community service	115 (1.55)
Other	407 (5.50)
Missing	0
Duration of leg oedema	
3 months - 1 year	767 (10.51)
1–2 years	723 (9.91)
2–5 years	1527 (20.93)
>5–10 years	1669 (22.88)
>10 years	2609 (35.76)
Missing	102
Mobility	
Normal	4138 (56.01)
Walking with aid	2400 (32.49)
Chair bound	689 (9.33)
Bedbound	161 (2.18)
Missing	9
Unilateral leg oedema	1821 (24.62)
Bilateral leg oedema	5576 (75.38)
Missing	0
Concomitant disease	
Diabetes	1382 (18.68)
Missing	0
Heart failure/ ischemic heart disease	1160 (15.68)
Missing	0
Peripheral arterial disease	242 (3.27)
Missing	0
Neurological disease	658 (8.92)
Missing	18
Leg wound	
Absent	6521 (88.36)
Present	859 (11.64)
Missing	17
Cellulitis within 12 months	
Absent	6151 (84.24)
Present	1151 (15.76)
Missing	95
Classification of chronic oedema	
Primary lymphoedema	1378 (19.03)
Secondary lymphoedema	5863 (80.97)
Missing	156
Related to cancer or its treatment	1021 (17.48)
Non-cancer	4821 (82.52)
Missing	21
Venous disease	2366 (49.16)
Immobility	1792 (37.23)
Obesity	1477 (30.69)
Filariasis	11 (0.23)
Missing	8
Unilateral leg oedema	1821 (24.62)
Bilateral leg oedema	5576 (75.38)
Missing	0
ISL scale^a^ (*n* = 917)	
I	211 (23.01)
II	526 (57.36)
III	180 (19.63)
Missing	1
Treatment with compression therapy	
Compression garment	5036 (69.25)
Multilayer bandage	1854 (25.50)
Compression wrap	630 (8.66)
At least one of the above	5692 (78.27)
No compression	1580 (21.73)
Missing	150
Good control of swelling	4243 (62.26)
Missing	582^b^

^a^The ISL stage tool has the following: Stage I: Early onset of the condition, with a collection of tissue oedema that decreases with limb elevation. The oedema may be pitting. Stage II: Limb elevation alone rarely reduces swelling and pitting is manifested. Stage III: The tissue is fibrotic (hard) and pitting is absent. Skin changes such as thickening, hyperpigmentation, increased skin folds, fat deposits, and warty overgrowths develop.

^b^Including cases either classified as ”unsure ”or ”not known”.

### Treatment with compression therapy

Of the total group the majority received compression therapy (78.3%). Good control of swelling was judged by the clinician in 62.3% (4243/6815). Type of treatments are presented in Table 2.

### Characterization of patients with obesity (univariate analysis)

Eighteen factors were found to be significantly associated with increased weight categories in patients with chronic leg oedema, Table 3. *Increased weight category was positively associated with*: longer swelling duration, decreased leg mobility, diabetes, presence of a leg wound, cellulitis within 12 months (e.g. affecting 13.7% in the normal weight, 15.9% in class I-II obesity and 19.8% in class III obesity), secondary lymphoedema, non-cancer lymphoedema, cancer treatment, oedema due to immobility, obesity, or filariasis, having bilateral lymphoedema (compared to unilateral), treatment in specialized lymphoedema clinics, and receiving compression therapy. *Increased weight category was inversely associated with*: age, peripheral arterial disease, metastatic cancer and oedema due to venous disease.

**Table 3:** Univariate analysis of obesity status with chronic leg oedema (*n* = 7397).

	Normal weight (BMI 20–30)		Class I and II obesity (BMI 30–40)		Class III obesity (BMI > 40)		z-score^a^	*p*-value
	*N*	%	*N*	%	*N*	%		
Gender (*n* = 7396)								
Female	2224	69.39	1908	72.30	1082	69.72		
Male	1016	30.99	734	27.76	471	30.31	−1.01	0.31
Age (*n* = 7395)								
<45 years	500	15.60	247	9.36	171	11.02		
45–64 years	803	25.05	820	31.08	680	43.81		
65–74 years	659	20.56	676	25.63	408	26.29	−10.30	<0.001
75–84 years	705	22.00	665	25.21	250	16.11		
85+ years	538	16.79	230	8.72	43	2.77		
Oedema duration (*n* = 7295)								
<1 year	511	16.39	202	7.68	54	3.49		
1–2 years	430	13.79	216	8.22	77	4.97		
2–5 years	628	20.14	592	22.52	307	19.83	17.20	<0.001
5–10 years	567	18.18	618	23.51	484	31.27		
>10 years	982	31.49	1001	38.08	626	40.44		
Leg mobility (*n* = 7388)								
Walks unaided	2004	62.59	1423	53.96	711	45.90		
Walks with aid	806	25.17	947	35.91	647	41.77	5.53	<0.001
Chair bound	270	8.43	239	9.06	180	11.62		
Bedbound	122	3.81	28	1.06	11	0.71		
Diabetes (*n* = 7397)								
Absent	2871	89.55	2054	77.83	1090	70.23		
Present	335	10.45	585	22.17	462	29.77	16.81	<0.001
Heart failure/Ischemic heart disease (*n* = 7397)								
Absent	2722	84.90	2202	83.44	1313	84.60		
Present	484	15.10	437	16.56	239	15.40	0.73	0.46
Peripheral arterial disease (*n* = 7397)								
Absent	3089	96.35	2542	96.32	1524	98.20		
Present	117	3.65	97	3.68	28	1.80	−2.66	0.008
Neurological disease (*n* = 7379)								
Absent	2910	91.08	2361	89.67	1450	93.49		
Present	285	8.92	272	10.33	101	6.51	−1.58	0.11
Presence of a leg wound (*n* = 7380)								
Absent	2838	88.85	2343	88.92	1340	86.40		
Present	356	11.15	292	11.08	211	13.60	2.03	0.043
Cellulitis within 12 months (*n* = 7302)								
Absent	2694	86.35	2213	84.11	1244	80.21		
Present	426	13.65	418	15.89	307	19.79	5.26	<0.001
Classification (*n* = 7241)								
Primary	741	23.83	469	18.00	168	11.02		
Secondary	2369	76.17	2137	82.00	1357	88.98	10.35	<0.001
Secondary cause (*n* = 5842)								
Cancer	653	27.80	300	14.04	68	5.01		
Non-cancer	1696	72.20	1836	85.96	1289	94.99	17.92	<0.001
Cancer treatment (*n* = 1016)								
Absent	89	13.73	23	7.67	10	14.71		
Present	559	86.27	277	92.33	58	85.29	1.96	0.05
Metastatic cancer (*n* = 1016)								
Absent	573	88.43	282	94.00	61	89.71		
Present	75	11.57	18	6.00	7	10.29	−2.25	0.025
Non-cancer cause of swelling (*n* = 4813)								
Venous								
Absent	774	45.74	909	49.59	764	59.32		
Present	918	54.26	924	50.41	524	40.68	−7.12	<0.001
Immobility								
Absent	1133	66.96	1120	61.10	768	59.63		
Present	559	33.04	713	38.90	520	40.37	4.29	<0.001
Obesity								
Absent	1680	99.29	1217	66.39	439	34.08		
Present	12	0.71	616	33.61	849	65.92	36.41	<0.001
Lymphatic filariasis								
Absent	1690	99.88	1831	99.89	1281	99.46		
Present	2	0.12	2	0.11	7	0.54	2.30	0.021
Limbs affected								
unilateral	1180	36.81	507	19.21	134	8.63		
bilateral	2026	63.19	2132	80.79	1418	91.37	22.22	<0.001
Facility (*n* = 7397)								
Specialist LD	2129	66.41	2116	80.18	1371	88.34		
General acute	709	22.11	413	15.65	137	8.83	−13.13	<0.001
Community	70	2.18	42	1.59	3	0.19	−5.78	<0.001
Other/unknown	298	9.30	68	2.58	41	2.64	−12.74	<0.001
Compression treatment								
Absent	875	28.13	524	20.04	181	1171		
Present	2236	71.87	2091	79.96	1365	88.29	12.86	<0.001
Control of swelling								
No	1097	37.96	942	38.45	533	36.14		
Yes	1793	62.04	1508	61.55	942	63.86	0.86	0.39

^a^A positive z-score means a positive association, a negative score is an inverse association.

### Characterization of patients with obesity (multivariate analysis)

The following ten factors remained independently associated with increased weight categories: diabetes (OR 2.4), secondary lymphoedema (OR 2.7), cellulitis within 12 months (OR 1.2), bilateral lymphoedema (OR 3.6), compression therapy (OR 2.1), increased swelling duration (reference being <1 year; 1–2 years OR 1.3, 2–5 years OR 2.5, 5–10 years OR 3.6, >10 years OR 3.5) decreased mobility (walking with aid OR 1.9, being chair bound OR 1.2) and age (reference being <45 years; being 45–64 years OR 1.5, 75–84 years OR 0.6, being 85+ years OR 0.2). Patient’s with increased weight categories had significantly lower presentation of peripheral arterial disease (OR 0.7) and poorer control of chronic oedema (OR 0.8) compared to normal weight. Detailed results are reported in Table 4.

**Table 4:** Ordinal logistic regression analysis: independent factors associated with level of obesity in patients with chronic leg oedema (*n* = 6706).

Factor	Odds ratio	95% CI	z-score	*p*-value
Diabetes				
Absent	1.000			
Present	2.412	2.137, 2.722	14.26	<0.001
Age				
<45 years	1.000			
45–64 years	1.532	1.297, 1.811		
65–74 years	0.916	0.766, 1.094		
75–84 years	0.574	0.476, 0.691	−17.03	<0.001
85+ years	0.190	0.150, 0.239		
Swelling duration				
<1 year	1.000			
1–2 years	1.328	1.052, 1.677		
2–5 years	2.519	2.062, 3.077	15.11	<0.001
5–10 years	3.596	2.947, 4.387		
>10 years	3.484	2.867, 4.234		
Mobility				
Walks freely	1.000			
Walks with aid	1.947	1.735, 2.186		
Chair bound	1.196	1.002, 1.472	4.88	<0.001
Bedbound	0.878	0.533, 1.447		
Classification of chronic oedema				
Primary	1.000			
Secondary	2.699	2.354, 3.096	14.21	<0.001
Cellulitis < 12 months				
Absent	1.000			
Present	1.199	1.053, 1.364	2.75	0.006
Peripheral arterial disease				
Absent	1.000			
Present	0.711	0.545, 0.928	−2.51	0.012
Control of swelling				
Absent	1.000			
Present	0.825	0.740, 0.920	−3.47	0.001
Bilateral swelling				
Absent	1.000			
Present	3.559	3.149, 4.021	20.35	<0.001
Compression use				
Absent	1.000			
Present	2.074	1.814, 2.372	10.66	<0.001

### Severity of chronic oedema/lymphoedema (univariate analysis)

In a sub-group of patients (*n* = 918) the severity of chronic oedema was also assessed, Table 5. With increased weight categories, the skin was significantly more likely to be non-pitting, more hard/fibrotic and Stemmer’s sign was more often positive. Patients with increased weight had more advanced stages of chronic oedema when assessed by ISL-staging, e.g. 13.8% of patients being normal weight had ISL stage III chronic oedema, increasing to 17.8% in patients with class I or II obesity and 38.5% in the class III obesity.

**Table 5:** Obesity and leg swelling: Chronic oedema factors (*n* = 918).

	Normal weight (BMI 20–30)		Class I and II obesity (BMI 30–40)		Class III obesity (BMI > 40)		z-score
	*N*	%	*N*	%	*N*	%	*p*-value
Pitting							
Non pitting	107	25.54	104	30.77	65	40.37	−3.35
Pitting	312	74.46	234	69.23	96	59.63	0.001
Tissue quality							
Soft	297	70.88	247	73.08	85	52.80	2.97
Hard (fibrotic)	122	29.12	91	26.92	76	47.20	0.003
Stemmer’s sign							
Negative	175	41.77	142	42.01	44	27.33	2.39
Positive	244	58.23	196	57.99	117	72.67	0.017
ISL scale^a^							
Stage I	118	28.16	85	25.22	8	4.97	6.62
Stage II	243	58.00	192	56.97	91	56.52	<0.001
Stage III	58	13.84	60	17.80	62	38.51	

^a^The ISL stage has the following: Stage I: Early onset of the condition, with a collection of tissue oedema that decreases with limb elevation. The oedema may be pitting. Stage II: Limb elevation alone rarely reduces swelling and pitting is manifested. Stage III: The tissue is fibrotic (hard) and pitting is absent. Skin changes such as thickening, hyperpigmentation, increased skin folds, fat deposits, and warty overgrowths develop.

### Quality of life

Increased weight categories was significantly associated with reduced function, impact on appearance and more severe symptoms, but not emotion assessed by LYMQOL. However, the overall LYMQOL score was not significantly different, Table 6. EuroQol and EQ-VAS scores demonstrated significantly lower quality of life with increased weight, compared to those of normal weight.

**Table 6:** Quality of life by obesity class; LYMQOL & EuroQol^a^.

	*N*	Mean	SD	F(df)	*p*-value
LYMQOL					
Function					
Normal weight	236	16.71	6.55		
Class I and II obesity	161	18.01	6.75	10.70 (2, 462)	<0.001
Class III obesity	68	20.88	6.43		
Appearance					
Normal weight	236	14.62	6.21		
Class I and II obesity	161	15.96	6.73	5.60 (2, 462)	0.002
Class III obesity	68	17.35	5.73		
Symptoms					
Normal weight	236	10.24	3.86		
Class I and II obesity	161	10.88	3.91	8.82 (2, 462)	<0.001
Class III obesity	68	12.54	4.68		
Emotion					
Normal weight	236	11.14	4.58		
Class I and II obesity	161	10.99	4.55	1.78 (2, 462)	0.17
Class III obesity	68	12.22	5.29		
Overall					
Normal weight	235	6.09	2.15		
Class I and II obesity	160	5.85	2.29	1.10 (2, 460)	0.33
Class III obesity	68	5.69	2.21		
Euroqol (EQ-5D)					
Normal weight	236	0.576	0.363		
Class I and II obesity	160	0.503	0.373	5.00 (2, 461)	0.007
Class III obesity	68	0.427	0.370		
EQ-VAS					
Normal weight	236	62.4	21.5		
Class I and II obesity	160	57.7	21.8	4.92 (2, 461)	0.008
Class III obesity	68	54.9	19.7		

Low values of LYMQOL indicate less impact on the patient, with higher scores demonstrating greater impact. The assessed overall quality of life in LymQol goes from zero (worst health) to 10 (best health). Euroqol (EQ-5D) gives a score 0 (death) to 1.00 (perfect health). EQ-VAS ranges from 0 to 100; 0 means the worst health the patient can imagine and 100 means perfect health.

^a^Normal weight (BMI 20–30); class I and II obesity (BMI 30–40), class III obesity (BMI >40).

## Discussion

The main results from this study show that more than half of the patients with chronic leg oedema/lymphoedema are affected by obesity, and the severity of oedema increases with each weight category. The most advanced stage of lymphoedema with fibrotic tissue (ISL III) affected 14% of patients with normal weight, 18% with class I-II obesity, and 39% with class III obesity (*p* = 0.001). The clinical observation of the challenges of managing chronic oedema in obesity was confirmed, with fewer patients reaching good oedema control compared to normal weight. These findings build on previous research reporting the unfavorable influence of obesity on the lymphatics and oedema management [2–4, 6, 8, 16, 21], with the advantage of being a large multicenter international study involving over 7000 clinically evaluated patients.

Obesity-induced oedema is a newly recognized condition, described by some to affect those at a BMI over 40. With increased BMI the oedema worsens and at BMI > 60 lymphoedema is almost inevitable, when determined by lymphoscintigraphy [22]. However, clinical obesity guidelines/comorbidity studies rarely mention lymphoedema as a complication [23–26] or suggest it as a standard part of a physical examination [27]. The prevalence of obesity-induced lymphoedema is yet to be determined but could be expected to be high, taking the above mentioned findings into account together with the notion that obesity affects every third US citizen and 8% of the population have a BMI ≥ 40 [28]. Increased awareness of this complication may increase the chance of early diagnosis and treatment, before the development of advanced lymphoedema – preventing complications such as wounds and bacterial cellulitis. The identification of chronic oedema can be performed at the bedside or in the patient’s home, with either the pitting oedema test or a positive Stemmer’s sign (see methods for how this is performed), used in this study. If in doubt, a lymphoscintigraphy can be performed.

In the study we aimed to investigate the impact/association of obesity on the characteristics of people with chronic oedema. People with increased obesity class had decreased mobility (walking with aids or being chair bound), which by itself contributes to oedema development due to gravitational forces and less activation of the calf muscle pump. Importantly, the risk of cellulitis/erysipelas within 12 months increased with each weight category, affecting every fifth patient with class III obesity. Similar findings have been reported by others [4, 21], including our previous publication [29]. Surprisingly, a significantly lower presentation of peripheral arterial disease (PAD) was seen with increased weight. The diagnosis of PAD was made by the investigator without formal definitions. Therefore, we cannot exclude that this finding may be biased by oedema/obesity itself, masking the presence of peripheral palpable foot pulses (if this was used for the diagnosis).

The high prevalence of advanced lymphoedema in obesity, identified in this and other studies [4, 8] may explain why we found a high accumulation of these patients in specialist lymphoedema services. Our finding that compression usage was associated with increased weight, is likely a reflection that these patients have more advanced forms of lymphoedema and are recruited from specialist services who routinely use compression in all patients. Less surprisingly, increased weight was associated with diabetes, secondary lymphoedema, bilateral swelling, and increased swelling duration. All these factors may explain the lower quality of life with increased weight, identified by both a disease-specific and a general quality of life tool. The level of class III obesity varied greatly among the countries ranging from 1% in Italy to 47% in Canada, this is likely to reflect the site of recruitment but may also reflect the different level of obesity among countries.

There is a gap in our knowledge surrounding why obesity causes lymphoedema [13, 22, 30]. One hypothesis is that the lymphatics are normal but the production of lymph is increased, overwhelming the lymphatic system. Compressed lymphatic vessels impacting lymphatic drainage due to pressure from the weight, has also been suggested [3]. Other theories involve inflammation from adipose tissue causing lymphatic destruction [22]. Studies of mice with obesity show reduced lymphatic pumping capacity, decreased lymphatic vessel density, increased lymphatic leakiness and infiltration of inflammatory cells around lymphatics compared to normal weight [31, 32]. Conversely, stagnant lymphatic fluid also stimulates adipogenesis. Animal models show lipid accumulation by lymphstasis, being associated with an upregulation of adiponectin and adipogenic transcription factors [7, 12]. These lipid factors, including free fatty acids, have adipogenic properties in vitro [33]. Inflammation may also play a part stimulating adipogenesis, e.g. T-cell inhibition/depletion of CD4+ cells decrease the severity of lymphoedema in animals with obesity [9, 34, 35]. The human lymphoedema fat tissue appear different compared to normal adipose tissue, with 24% larger [36] and highly variable adipocytes in size [10] compared to adipose tissue. Larger lobules, more collagen and a decreased number of M2 macrophages is also seen in lymphoedema adipose tissue [10]. As obesity may damage the lymphatics, and stagnant lymph may stimulate adipogenesis, a vicious loop has been created [4].

The management of obesity-induced lymphoedema is largely based on expert opinion and involves good skin care to ensure an intact skin barrier, compression, weight loss and exercise. These patients are therefore treated like most other lymphoedema patients receiving complex decongestive therapy (CDT) [4, 17] – but there are many challenges. Due to the deep skin folds the skin may be compromised, wet and therefore often affected by mycosis. Applying a therapeutic level of compression is also challenging, especially in those with ISL stage III, with fibroadipose tissue. The level of compression is effected by the increased size of the limb and it is complex to apply in those with deep skin folds and limb shape distortions [37]. Some patients develop massive localized lymphoedema, with large lobules, frequently affecting the thigh, that are difficult to apply compression to. A lot of padding may be required to prevent pressure injuries in a limb with severe shape distortion such as this. The effect of increased padding causes a large limb circumference in which applying a therapeutic level of pressure is challenging. To achieve compression the application technique must be adapted which includes using more layers of bandage. The cumulative effect is a bulky limb which in turn reduces mobility and limb function and may cause difficulty in maintaining personal hygiene. Furthermore, the oedema may be reaching the genitals and abdomen, and in reality, these areas cannot be easily compressed. Full leg compression may itself move fluid into the genital area thus compounding the problem. Comorbidities such as severe heart failure may also limit the compression, due to the risk of complications such as pulmonary oedema. Severe diabetic neuropathy may likewise increase the risk of pressure injuries from compression, and polypharmacy is also an issue with calcium channel blockers aggravating oedema. While compression bandages lead to one problem a second is the transition to compression garments used to maintain the effects of the CDT. Finding a correct type of garment is associated with many challenges and may require a combination of hosiery and compression wraps to prevent rebound oedema. While the clinical presentation of the swelling may indicate that a full leg garment or tights are required this may be practically impossible to achieve. Due to obesity, some patients may not be able to apply or remove the garments themselves or to perform exercise. All these factors may explain why control of swelling was less likely to be achieved in our patients with higher weight categories (OR 0.8). Understanding the quality of life and psychosocial impact of these patients (e.g. living alone, not being able to walk, maintaining personal hygiene, addressing pain) needs to be integrated into the treatments plans to enable improved adherence. Clinicians may be obsessed with limb volume reduction, whereas the patient priorities may be completely different. Although compression therapy is central to alleviating symptoms and complications, chronic oedema/lymphoedema is often a symptom/consequence of severe obesity. Addressing the real cause of the problem is the obesity issue. Weight loss would seem intuitive to prevent further lymphatic damage and/or to reverse the lymphatic function but would need further confirmation in clinical studies. Studies of mice with obesity have shown that diet-induced weight loss can reverse the lymphatic damage [32], including aerobic exercise that is independent of weight loss [31]. Preliminary results in humans have suggested that if BMI is reduced below 50 there is a 50% chance that the lymphatic dysfunction is normalized on lymphscintrography [17]. A meta-analysis of four RCT’s with patients with breast-cancer-related lymphoedema found that weight loss decreased the volume of both arms (affected by lymphoedema and not) but the treatment did not reduce the severity of lymphoedema as determined by interlimb difference in arm volume. The authors concluded that effect of weight loss as a preventative measure needs to be investigated [38]. The effect of bariatric surgery on limb volumes is promising but based on case-series [39]. Taken together, a collaboration between lymphoedema services and a bariatric weight loss center may prove to be essential [17]. Future encouraging treatment modalities include an evaluation of new weight loss drugs.

Limitations to our study include the following: 1. Weight categories were classified as either normal weight, class I-II obesity or class III obesity, using BMI when this was available. This was a pragmatic decision based on the complexity of the different study sites, including community care when e.g. weight scales are not available or patients are not able to stand up. Also, patients with overweight were included in the normal weight category which may have influenced the effect on increased weight categories. 2. Chronic oedema/lymphoedema was diagnosed with either a positive pitting oedema test and/or Stemmer’s sign. Stemmer’s sign holds high sensitivity and specificity for the diagnosis of lymphoedema (reference being lymphscintrography), but may produce false positive results in obesity [40]. As the majority of the patients with class III obesity were mainly recruited from specialized lymphoedema centers, we feel confident that these patients had true chronic oedema/lymphoedema. 3. Extrapolation of the results to a more general population cannot be concluded from this data, due to the dominant recruitment from specialist lymphoedema services.

To conclude, the results from this study confirm the negative impact of obesity, and especially class III obesity, on chronic oedema/lymphoedema. More than half of the patients with chronic leg oedema have concomitant obesity, and the severity of oedema is worsened with increasing weight. Likewise, lymphoedema-related complications such as cellulitis/erysipelas is increased in patients with obesity. Despite these and previous findings, clinical guidelines rarely mention lymphoedema as a comorbidity to obesity. Future studies would need to assess the prevalence and impact of each weight category on chronic oedema, and whether weight loss interventions can prevent and/or reverse the lymphatic damage.

## Data Availability

All data generated or analyzed during this study are included in this published article.
